# Neurotrophic Effects of a Cyanine Dye via the PI3K-Akt Pathway: Attenuation of Motor Discoordination and Neurodegeneration in an Ataxic Animal Model

**DOI:** 10.1371/journal.pone.0017137

**Published:** 2011-02-11

**Authors:** Hitomi Ohta, Shigeyuki Arai, Kenji Akita, Tsunetaka Ohta, Shigeharu Fukuda

**Affiliations:** Research Center, Biomedical Institute, Hayashibara Biochemical Laboratories, Inc., Okayama, Japan; National Institute on Aging Intramural Research Program, United States of America

## Abstract

**Background:**

Neurotrophic factors may be future therapeutic agents for neurodegenerative disease. In the screening of biologically active molecules for neurotrophic potency, we found that a photosensitizing cyanine dye, NK-4, had remarkable neurotrophic activities and was a potent radical scavenger.

**Methodology/Principal Findings:**

In this study, we evaluated the effect of NK-4 on the protection of neurons against oxidative damage and investigated the associated intracellular signaling pathways. Subsequently, we evaluated the effect of NK-4 in an animal model of neurodegeneration. *In vitro*, NK-4 showed dose-dependent protection of PC12 cells from toxicity induced by oxidative stress caused by hydrogen peroxide (H_2_O_2_) or 6-hydroxydopamine (6-OHDA). Comparison of extracellular signal-regulated kinase signaling pathways between treatment with NK-4 and nerve growth factor (NGF) using K252a, an inhibitor of the NGF receptor TrkA, revealed that NK-4 activity occurs independently of NGF receptors. LY294002, a phosphatidylinositol 3-kinase (PI3K) inhibitor, blocked the protective effect of NK-4, and NK-4 caused activation of Akt/protein kinase B, a downstream effector of PI3K. These results suggest that the neuroprotective effects of NK-4 are mediated by the PI3K-Akt signaling pathway. NK-4 treatment also attenuated stress-induced activation of SAPK/JNK, which suggests that NK-4 activates a survival signaling pathway and inhibits stress-activated apoptotic pathways independently of the TrkA receptor in neuronal cells. *In vivo*, administration of NK-4 improved motor coordination in genetic ataxic hamsters, as assessed by rota-rod testing. Histological analysis showed that cerebellar atrophy was significantly attenuated by NK-4 treatment. Notably, the Purkinje cell count in the treated group was threefold higher than that in the vehicle group.

**Conclusions/Significance:**

These results suggest that NK-4 is a potential agent for therapy for neurodegenerative disorders based on the activation of survival signaling pathways.

## Introduction

Neurodegenerative diseases including Alzheimer's disease (AD), Parkinson's disease (PD), and motor neuron diseases are an enormous growing public health burden globally. Thus, there is a clear need to develop therapies that will halt or reverse their progression. Neurotrophic factors may serve as therapeutic agents because they play key roles in coordination of brain development and maintenance of brain function in adulthood. Indeed, the use of neurotrophic factors such as nerve growth factor (NGF) and brain derived neurotrophic factor (BDNF) for treatment of neurodegenerative diseases is widely advocated, based mainly on *in vitro* observations and preliminary success in animal models of AD, PD, Huntington disease, Rett syndrome, traumatic brain injury, and aging [1]–[6]. However, several properties limit the therapeutic application of neurotrophic factors. Their large size causes poor blood brain barrier penetration [7], and high antigenicity may lead to failure of long term efficacy. NGF and BDNF also interact at low affinity with the P75 receptor, and this might contribute to promotion of pain and other undesirable effects [8]. Therefore, small molecules are required as therapeutic agents that have high and long-lasting neurotrophic potency and do not cause serious adverse effects.

Therapies that promote cellular events including proliferation, differentiation, and migration, neurite-outgrowth, synaptogenesis, and myelination of neurons are needed for many neurodegerative diseases. Proliferation and growth of axonal and dendritic processes (neurites) are critical determinants of neuronal function, and disruption of these processes can lead to neuronal dysfunction. The PC12 cell line has been widely used in neurobiology to evaluate chemical effects on neurite-outgrowth [9]–[11]. Following exposure to neurotrophic factors such as NGF, PC12 cells proliferate and differentiate into a sympathetic neuron-like morphology, and develop extensive neuritic processes in a concentration-dependent manner [12]. Thus, we screened biologically active molecules for neurotrophic potency using PC12 cells, and we found that certain photosensitizing cyanine dyes have remarkable neurotrophic activities.

Cyanine dyes have been studied for over 150 years and continue to be of interest in biology and medicine [13]. One of these dyes, NK-4, has a variety of biological activities, including antimicrobial [14], macrophage-activating [15] and anticancer [16] properties, and is used practically as an immunomodulator in treatment with antiviral and anticancer agents [17]. However, the effect of cyanine dyes on neurodegenerative disease is not well established. Recently, we identified NK-4 as the most potent radical scavenger among a series of cyanine dyes, and found that NK-4 was effective for treatment of cerebral ischemia [18]. Thus, we focused on NK-4 as an agent for treatment of neurodegeneration.

Tropomyosin-related kinases (Trks) are essential for neurotrophin family-mediated cellular events, including neuronal survival, differentiation, and synaptic function [19] via activation of downstream signaling mediators of phosphatidylinositol 3-kinase (PI3K) and the survival signaling kinase Akt [20]. In a therapeutic context, Akt has been shown to mediate striking neurotrophic and anti-apoptotic effects *in vivo* [21]. In contrast, in relation to neuronal degeneration, oxidative stress may cause activation of stress-activated protein kinase/c-Jun N-terminal kinase (SAPK/JNK). Inhibition of the SAPK/JNK pathway by activation of PI3K-Akt has been proposed as a potential therapeutic target in neurodegenerative diseases [22]. These observations suggest that PI3K-Akt is of particular therapeutic interest, and it is likely that therapeutic targets related to kinase signaling pathways will emerge.

Hamsters with inheritable ataxia, an autosomal recessive trait, were used to evaluate the preclinical efficacy of NK-4. This ataxic animal originally arose spontaneously in one of our breeding colonies, and shows typical clinical signs of ataxic gait, including a slight trembling of the head, unsteady walking, and stumbling after 7 weeks of age [23], [24]. These ataxic symptoms were well correlated with the cerebellum atrophy and Purkinje cells degeneration. The Purkinje cell degeneration of the mutant was characterized by suppression of Nna1, a gene discovered in an axonal regenerative context [23], [25]. Similarly, abnormal development of Purkinje cell dendrites in mice with Purkinje cell degeneration has been linked to a deletion mutant in exon 7 of Nna1 [26]. We refer to the ataxic mutant line as *hm*PCD (hamster PCD).

In this study, we demonstrate that NK-4 has potent neurotrophic activity and protects neuronal cells against toxicity, including oxidative stress, by activating the PI3K-Akt signaling pathway *in vitro*. We further show that administration of NK-4 to the ataxic hamster significantly improved motor discoordination, with reversal of damage to Purkinje cells and the cerebellum.

## Methods

### Ethics Statement

All animal protocols were approved by the Institutional Animal Care and Use Committee of Hayashibara Biochemical Laboratories (permit number F-902), and were conducted in accordance with the guidelines of the Care and Use of Laboratory Animals at Hayashibara Biochemical Laboratories.

### Chemicals

NK-4 (4,4′-[3-{2-(1-ethyl-4(1H)-quinolylidene)ethylidene}propenylene]bis(1- ethylquinolinium iodide)) was synthesized at Hayashibara Biochemical Laboratories, Inc. (Okayama, Japan). A stock solution of 6.3 mM NK-4 was prepared in DMSO (Sigma, St. Louis, MO) and stored at room temperature with protection from light. Just before use, the stock solution was diluted with DMSO to give a 32 µM solution. This solution was used for experiments with further dilution. NGF, K252a, and LY294002 were purchased from Wako Pure Chemical Industries (Osaka, Japan). Phospho-Akt (Ser 473) antibody, Phospho-SAPK/JNK (Thr183/Thr185) antibody, Akt antibody, and SAPK/JNK antibody were obtained from Cell Signaling Technology, Inc. (Beverly, MA), anti-calbindin D28K antibody was from Chemicon International, Inc. (Temecula, CA) and Alexa_488_-conjugated anti-rabbit IgG antibody was from Molecular Probes (Eugene, OR).

### Cell Culture

The rat pheochromocytoma cell line PC12 (Human Science Research Resources Bank, Osaka, Japan) was cultured in Dulbecco's Modified Eagle Medium (D-MEM; Nissui, Tokyo, Japan) supplemented with heat-inactivated 10% fetal bovine serum, 5% horse serum. For cell growth and neurite-outgrowth assays, PC12 cells were harvested using 0.1% (w/v) trypsin containing 0.03% (w/v) EDTA, and seeded at a density of 5,000 cells/100 µl in 96-well plates pre-coated with collagen type IV. After a 24-hr pre-culture, PC12 cells were exposed to NK-4. For neurite-outgrowth quantification, cells were treated with NK-4 at a low concentration of NGF (5 ng/ml). Cells were incubated for 72 hr prior to evaluation of cell growth and neurite-outgrowth. Cell survival was determined using alamarBlue dye (Trek Diagnostic Systems, Cleveland, OH) [27]. To measure neurite-outgrowth, cells were fixed with 4% glutaraldehyde in phosphate buffer and visualized by phase-contrast microscopy. Representative cells were photographed for each well. The percentage of cells with neurites that were at least twice the diameter of the cell body was calculated. Neurites were identified and counted from ∼100 cells per photograph. In some experiments a PI3K or Trk inhibitor, LY294002 or K252a, was added at the indicated concentration to serum-free medium and incubated for 15 min, after which 50 ng/ml NGF or 250 nM NK-4 with 5 ng/ml NGF was added.

### Evaluation of the protective effects of NK-4 against neurotoxic environments

Hydrogen peroxide (H_2_O_2_) and 6-hydroxydopamine (6-OHDA) were used to produce neurotoxic environments in which to investigate the cytoprotective effects of NK-4. PC12 cells were seeded at a density of 2×10^4^ cells/100 µl in 96-well plates pre-coated with collagen type IV and cultured for 24 hr prior to stimulation. To investigate the effect of NK-4 on H_2_O_2_- or 6-OHDA-induced cytotoxicity, PC12 cells were exposed to 200 µM H_2_O_2_ for 2 hr or 100 µM 6-OHDA for 24 hr in the presence or absence of NK-4. After treatment, cell survival was assessed using alamarBlue dye. All results were confirmed by visual inspection using phase-contrast microscopy.

### Western Blotting

After PC12 cells were treated for the indicated periods with NK-4, the culture medium was aspirated and cells were washed with ice-cold PBS. Subsequently, cells were homogenized with lysis buffer [62.5 mM Tris, pH 6.8, 150 mM NaCl, 0.02% (w/v) sodium azide, 2% (w/v) SDS, 10% glycerol, 50 mM DTT, 0.01% (w/v) bromophenol blue] and sonicated for 10 sec. Equal amounts of protein (20 µg) were separated on a SDS polyacrylamide gel and transferred to a nitrocellulose membrane (Osmonics, Inc., Gloucester, MA). Blots were blocked for 1 h at room temperature with 5% (w/v) nonfat dried milk in Tris-buffered saline (10 mM Tris, pH 7.6, and 150 mM NaCl) containing 0.1% Tween 20. The membrane was incubated overnight at 4°C with specific antibodies. Rabbit polyclonal antibodies against Akt, SAPK/JNK and their phosphorylated forms were used in the study. The blot was then incubated with the G-horseradish peroxidase conjugated anti-rabbit immunoglobulin (Dako, Glostrup, Denmark). Immunoreactive proteins were detected with the enhanced chemiluminescence Western blotting detection system. The relative density of the protein bands was quantified by densitometry using Image Master 2D (Amersham Pharmacia Biotec, Piscataway, NJ).

### Animals

Male and female inbred homozygous ataxic mutant Syrian hamsters (*Mesocricetus auratus*) and age- and sex-matched wild-type hamsters were used in the study [23], [24]. The animals were housed individually with nesting materials supplied, and food and water were provided *ad libitum*. NK-4 solution was injected intraperitoneally at a dose of 20 or 100 µg/kg once a day for 7 weeks, beginning at 3 weeks of age. Controls received 200 µl of saline.

### Behavioral analysis

Motor coordination of ataxic animals was determined in a rota-rod task performed every week after injection of NK-4. In this test, animals were placed in a rota-rod apparatus (6-cm diameter, Hayashibara Biochemical Laboratories, Inc.) with a constant rotation rate of 6 rpm. The apparatus consisted of a horizontal motor-driven rotating rod in which the animals were placed perpendicular to the long axis of the rod, with the head directed against the direction of rotation so that the hamster has to progress forward to avoid falling. The trial was stopped when the animal fell down or after a maximum of 180 s. The time spent in the rotating rod was recorded for each animal and trial. All animals received a pretraining session to familiarize them with the rotating apparatus one week before the first test session. Subsequently, six consecutive trials were performed for every animal in each session every week [28], [29]. The results of the sixth test were used in the statistical comparison. At the end of the study, each hamster was placed in a plastic cage (20×30×15 cm) and the spontaneous falling frequency was counted for 60 s [30].

### Volumetric measurements of hamster brain

Animals were deeply anesthetized with pentobarbital (50 mg/kg), brains were dissected and fixed with 10% buffered-formalin and pictured from the constant distance from the top. The sizes of the cerebellum were measured and the volumes were calculated as V = ab^2^/2, where “a” and “b” are the lengths of the major and minor axes of the cerebellum, respectively [31].

### Histology

Brains were embedded in paraffin, and then mid-sagittal sections of 5 µm thickness were stained with hematoxylin and eosin (H&E). To count the calbindin-positive cells, dewaxed and rehydrated paraffin sections were permeablized and blocked with PBS containing 0.2% Triton X-100 and 5% BSA. The sections were then incubated with anti-calbindin antibody (1∶500) 2 overnight at 4°C diluted in PBS containing 0.1% Triton X-100 and 2% BSA. After several washes in PBS, sections were incubated with Alexa_488_-conjugated secondary antibody (1∶50) [32]. Calbindin-positive cells in the Purkinje layer were counted as Purkinje cells. The density of granule cells was calculated in the tenth lobule of the cerebellum.

### Statistical Analysis

All values are expressed as means ± SD or SEM. A paired *t* test was used for comparison between 2 groups. One-way ANOVA with a subsequent Tukey-Kramer test was used to determine the significance of differences in multiple comparisons. Differences with a probability value of P<0.05 were considered to be significant.

## Results

### NK-4 promotes the growth and neurite-outgrowth of neuronal PC12 cells

**Figure 1:**
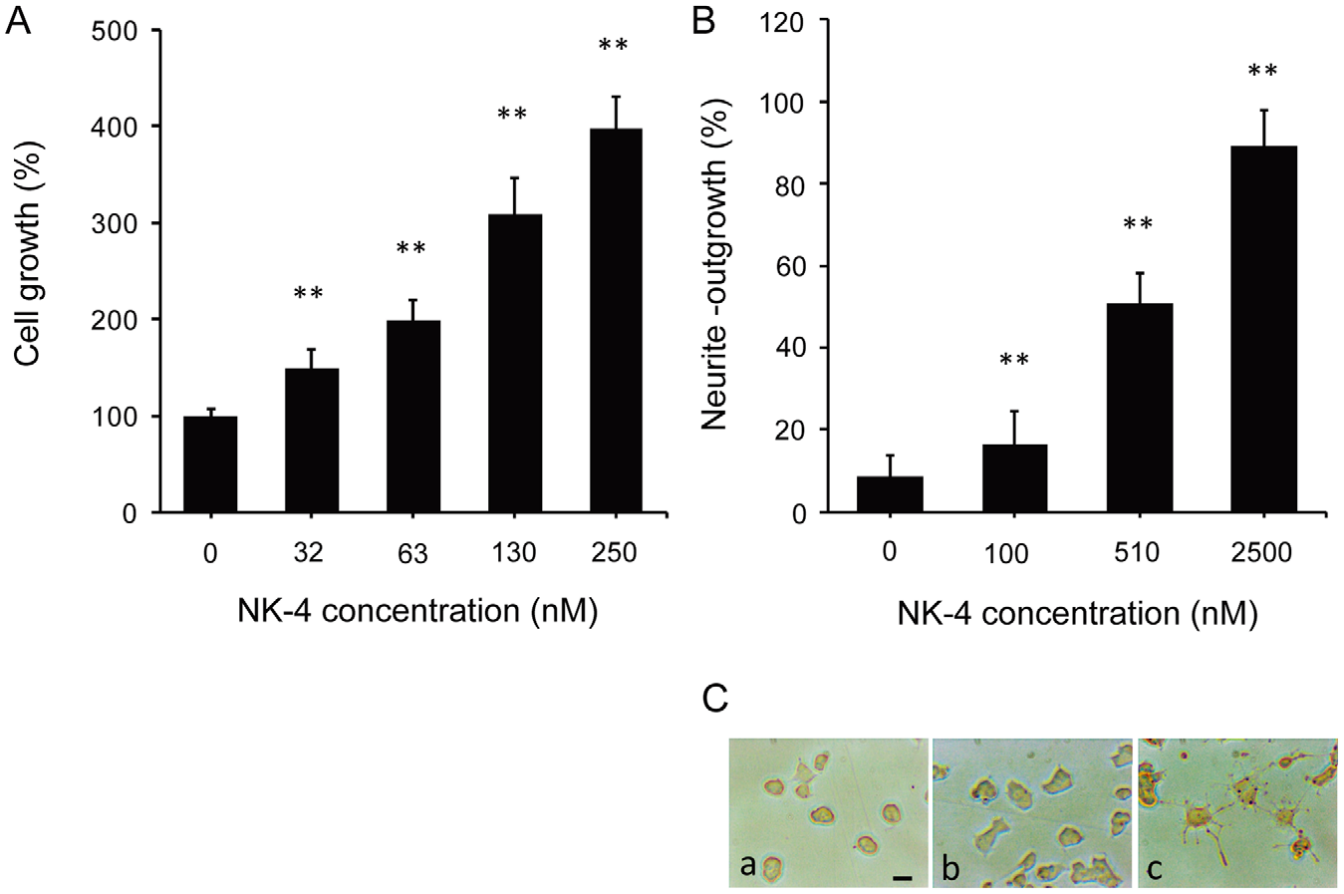
Neurotrophic effects of NK-4 in PC12 cells. (**A**) Effect of NK-4 on PC12 cell growth. PC12 cells were raised in D-MEM containing 10% FBS for 72 hr in the presence or absence of NK-4. The number of cells was assessed by alamarBlue assay. (**B**) Effect of NK-4 on NGF-primed neurite-outgrowth in PC12 cells. PC12 cells were treated with NGF (5 ng/ml) plus NK-4 (indicated concentrations) for 72 hr. The percentage of PC12 cells with neurites longer than twice the diameter of the cell body was calculated. Results are shown as a mean ± SD (n = 3). **P<0.01 vs. the respective control. (**C**) Phase contrast micrographs of PC12 cells. PC12 cells treated with NK-4 (1000 nM) plus NGF (5 ng/ml) (c) had longer neurites than those treated with NK-4 (1000 nM) (a) or NGF (5 ng/ml) (b) for 72 hr. Bar: 50 µm.

NK-4 induced PC12 cell growth (Fig. 1A) and promoted NGF-primed neurite extension (Fig. 1B), as shown by the appearance of neurite-outgrowth within 3 days (Fig. 1C). NK-4 alone promoted cell growth, but did not affect the morphological differentiation of PC12 cells. The effects of NK-4 were dose-dependent, based on fluorometric and quantitative analysis of cellular growth and neurite extension. The concentration at which NK-4 exerted its maximum effect differed when assessed by growth or neurite-outgrowth: a maximal effect was obtained at 250 nM as assessed by cell growth, whereas a higher dose of up to 2.5 µM was needed for neurite-outgrowth.

### Effects of NK-4 in PC12 cells treated with neurotoxins

**Figure 2:**
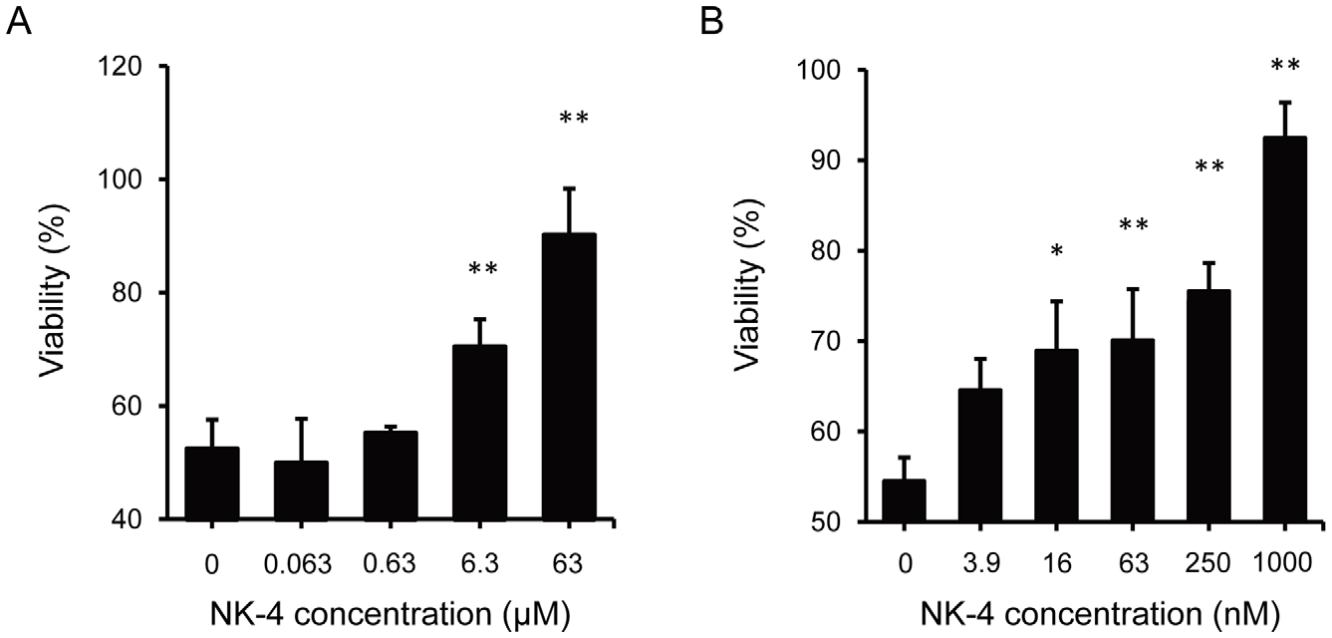
Cytoprotective effects of NK-4 against neurotoxins in PC12 cells. (**A**) Effect of NK-4 on H_2_O_2_-induced cytotoxicity. PC12 cells were treated with 200 µM H_2_O_2_ for 2 hr in the absence or presence of the indicated concentrations of NK-4. Control cells were incubated under the same conditions, but without H_2_O_2_. (**B**) Effect of NK-4 against 6-OHDA-induced toxicity. PC12 cells were treated with 100 µM 6-OHDA for 24 hr in the absence or presence of the indicated concentrations of NK-4. Control cells were incubated under the same conditions, but without 6-OHDA. All results were obtained by alamarBlue assay and are expressed as the percentage fluorescence of control cells. Values are means ± SD obtained in triplicate. *P<0.05, **P<0.01 for comparison of wells with and without NK-4.

The effect of NK-4 against oxidative toxicity induced by H_2_O_2_ was examined because neurons are vulnerable to the toxic effects of oxidative stress. In this model, the viability of cells exposed to 200 µM H_2_O_2_ for 2 hr was about 50% compared to controls without H_2_O_2_ exposure. NK-4 protected cells from H_2_O_2_ toxicity in a dose-dependent manner (Fig. 2A). Since oxidative stress induced by H_2_O_2_ occurred rapidly and severely, relatively high concentrations of NK-4 were required to obtain a significant protective effect. The cytoprotective effect of NK-4 was also found using 6-OHDA, a selective catecholaminergic neurotoxin that has been used to produce PD models *in vitro* and *in vivo* [33], [34]. When PC12 cells were cultured with 100 µM 6-OHDA for 24 hr, cell viability was significantly decreased to 60% compared with controls with no 6-OHDA (Fig. 2B). NK-4 dose-dependently attenuated cell damage induced by 6-OHDA and the results were significant at doses over 16 nM. We also found a protective effect of NK-4 against cell damage induced by serum deprivation (data not shown).

Given the cytoprotective effect of NK-4 against oxidative damage, we examined if NK-4 could directly scavenge H_2_O_2_, since it has been reported that H_2_O_2_ generated from 6-OHDA is involved in cytotoxicity [33]. NK-4 was able to detoxify reactive oxygen species (ROS) such as hydroxyl, superoxide and peroxy radicals [18], but showed no effect on H_2_O_2_ scavenging at concentrations up to 63 µM (data not shown).

### NK-4 does not act via Trks

**Figure 3:**
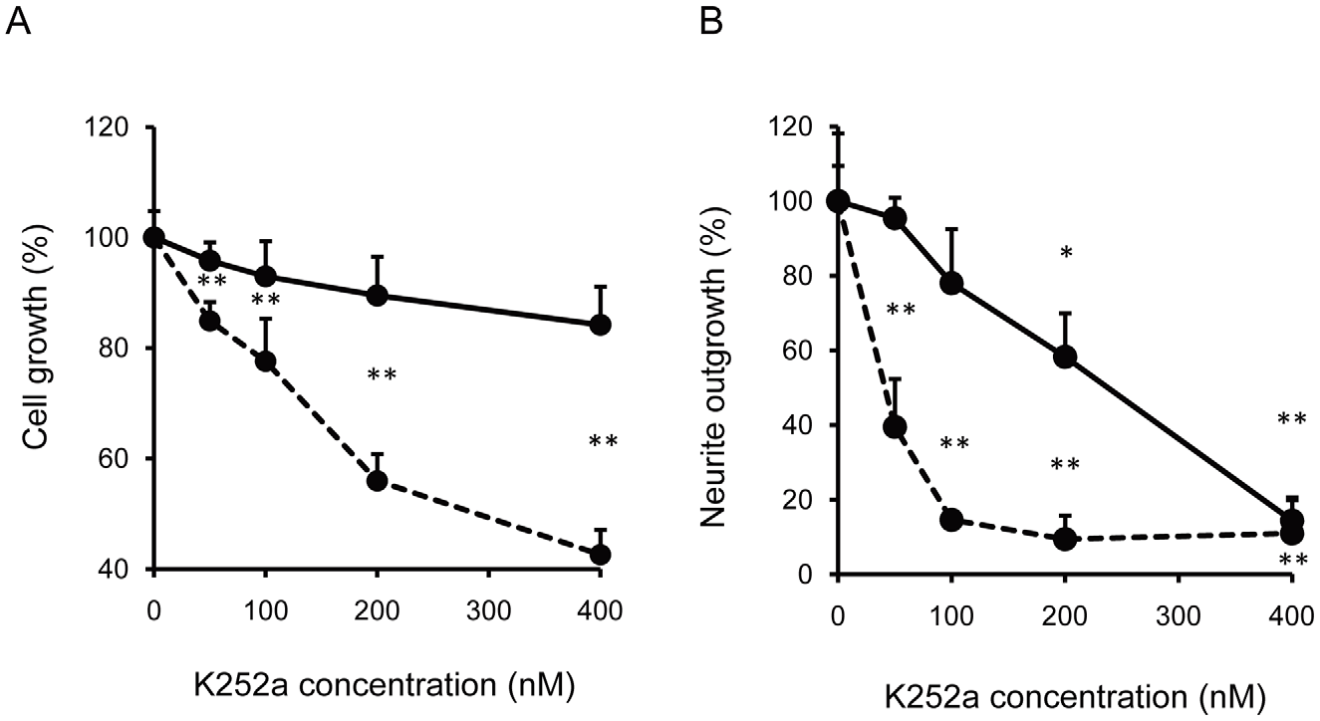
Effects of K252a, a Trk inhibitor, on PC12 cell growth (A) and neurite-outgrowth (B) induced by NK-4 (bold line) and/or NGF (broken line). PC12 cells were preincubated in serum-free D-MEM with the indicated concentrations of K252a for 15 min. NK-4 (250 nM) or NGF (50 ng/ml) was added and the cells were further incubated for 72 hr. In the neurite-outgrowth assay, 250 nM NK-4 with 5 ng/ml NGF, or 50 ng/ml of NGF alone was used. The number of cells was assessed by alamarBlue assay. The percentage of cells with neurites was quantified by phase contrast microscopy. Values are means ± SD in triplicate. **P<0.01 vs. control.

We next investigated if NK-4 acts via Trks to induce growth and neurite-outgrowth in PC12 cells. Trks includes the NGF receptor, Trk A, which mediates NGF-induced neuronal survival and differentiation in PC12 cells [35]. Pretreatment with K252a, a Trk inhibitor, dose-dependently inhibited NGF-induced cell growth, but did not inhibit that induced by NK-4 (Fig. 3A). This suggests that NK-4 activity occurs independently of TrkA activation in PC12 cells. Similarly, NGF-induced neurite-outgrowth was inhibited in a concentration-dependent manner by K252a, while the effect of NK-4 on neurite-outgrowth was not inhibited efficiently (Fig. 3B). A high K252a concentration did significantly decrease the effect of NK-4, which might be because neurite-outgrowth induced by NK-4 is dependent on the presence of a low level of NGF.

### Intracellular signaling events that underlie NK-4-mediated cytoprotection

**Figure 4:**
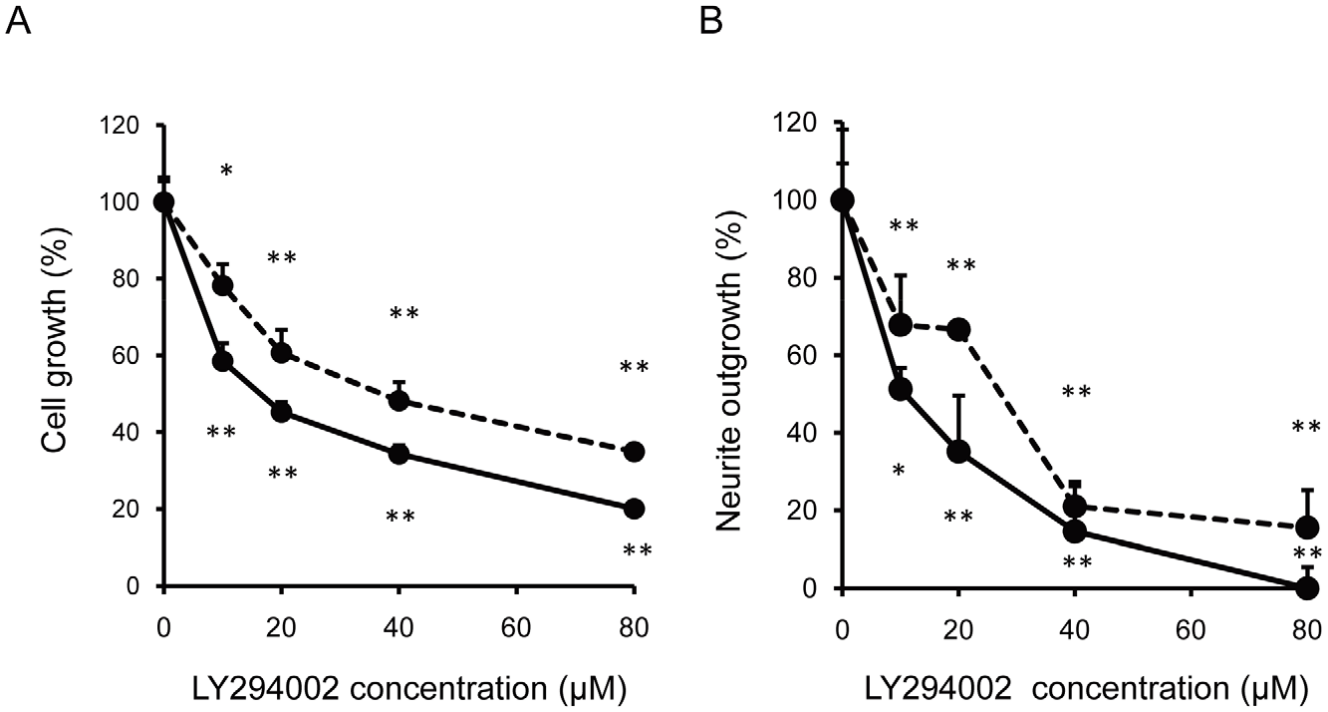
Promotion of PC12 cell growth and neurite-outgrowth through activation of PI3K by NK-4. Effects of LY294002, a PI3K inhibitor on cell growth (A) and neurite-outgrowth (B) induced by NK-4 (bold line) and/or NGF (broken line). PC12 cells were preincubated in serum-free D-MEM containing the indicated concentrations of LY294002 for 15 min. NK-4 (250 nM) or NGF (50 ng/ml) was added and the cells were further incubated for 72 hr. The number of cells was assessed by alamarBlue assay. In the neurite-outgrowth assay, 250 nM NK-4 with 5 ng/ml NGF, or 50 ng/ml of NGF alone was used. The percentage of cells with neurites was quantified by phase contrast microscopy. Values are means ± SD in triplicate. **P<0.01 vs. control.

To analyze the effect of NK-4 on survival and differentiation of PC12 cells further, we next examined whether NK-4 activates PI3K and its downstream signaling effector Akt. This cascade has been implicated in the survival signaling caused by NGF in serum-deprived PC12 cells, and in neuritogenesis in PC12 cells [36], [37]. We examined if LY294002, a specific PI3K inhibitor, blocked the activity of NK-4, and also determined the kinetics of Akt activation following NK-4 treatment of PC12 cells. LY294002 caused cell growth arrest of PC12 cells dose-dependently in the presence of NGF and NK-4 (Fig. 4A). An inhibitory effect of LY294002 was also found in the neurite-outgrowth assay (Fig. 4B). The neurotrophic effects of NK-4 and NGF on PC12 cells were similarly inhibited by the PI3K inhibitor.

**Figure 5:**
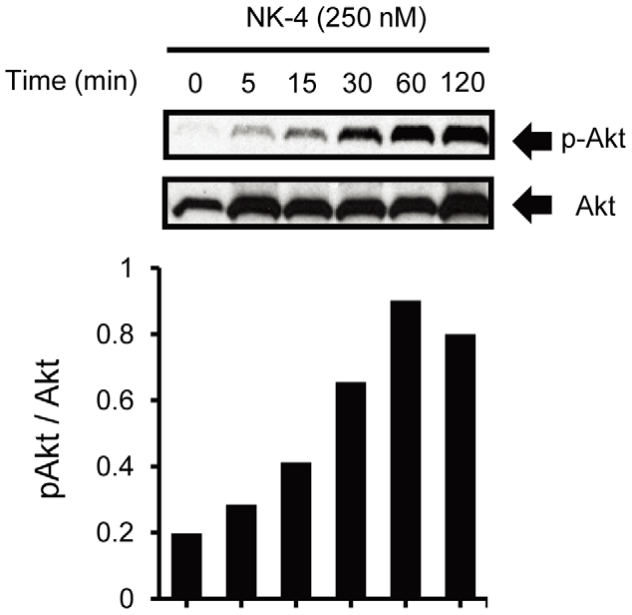
Induction of Akt phosphorylation by NK-4 in PC12 cells. PC12 cells were treated with 250 nM NK-4 for the indicated times. Whole cell lysates were analyzed by Western blotting using anti-phospho-Akt antibody (upper panel) or anti-Akt antibody (lower panel). The graph shows the ratio of phosphorylated Akt to total Akt at each time point.

Activation of Akt can be detected by Western blotting with antibodies that specifically recognize Akt phosphorylated at Ser 473, because this phosphorylation correlates with Akt activity [38]. As shown in Fig. 5, NK-4 induced phosphorylation of Akt in a time-dependent manner. Akt phosphorylation was detected by 5 min NK-4 treatment and maximally increased between 60 and 120 min. The dose of NK-4 required for induction of Akt phosphorylation was consistent with that required for promotion of PC12 cell growth. These results suggest that activation of PI3K and its downstream signaling effector Akt are important in NK-4-induced neurotrophic effects in PC12 cells.

### Effect of NK-4 on stress-activated SAPK/JNK

**Figure 6:**
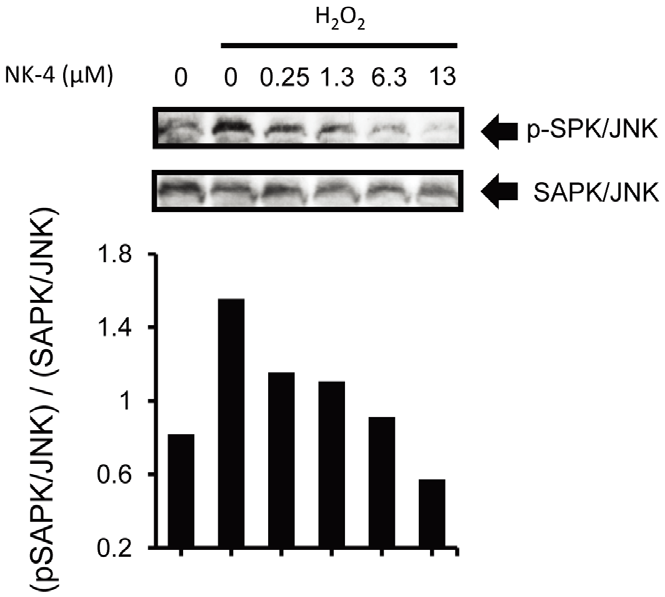
NK-4 attenuated H_2_O_2_-induced SAPK/JNK phosphorylation in PC12 cells. PC12 cells were treated with 400 µM H_2_O_2_ in the presence of the indicated concentrations of NK-4 for 2 hr. Whole cell lysates were analyzed by Western blotting using anti-phospho-SAPK/JNK antibody (upper panel), or anti-SAPK/JNK antibody (bottom panel). The graph shows the ratio of phosphorylated SAPK/JNK to total SAPK/JNK at each time point.

We subsequently investigated whether SAPK/JNK, a major cellular stress responsive protein induced by oxidative stress, plays an important role in NK-4-mediated effects in PC12 cells. PC12 cells treated with cytotoxic levels of H_2_O_2_ induced SAPK/JNK activation in PC12 cells (Fig. 6). This activation was attenuated by NK-4 in a concentration-dependent manner, and the dose of NK-4 was well correlated with the concentration found in the H_2_O_2_-induced cytotoxicity model in PC12 cells. This observation suggests that NK-4 might attenuate H_2_O_2_-induced cell death through inhibition of SAPK/JNK activation.

### Cerebellar ataxia in mutant hamsters

**Figure 7:**
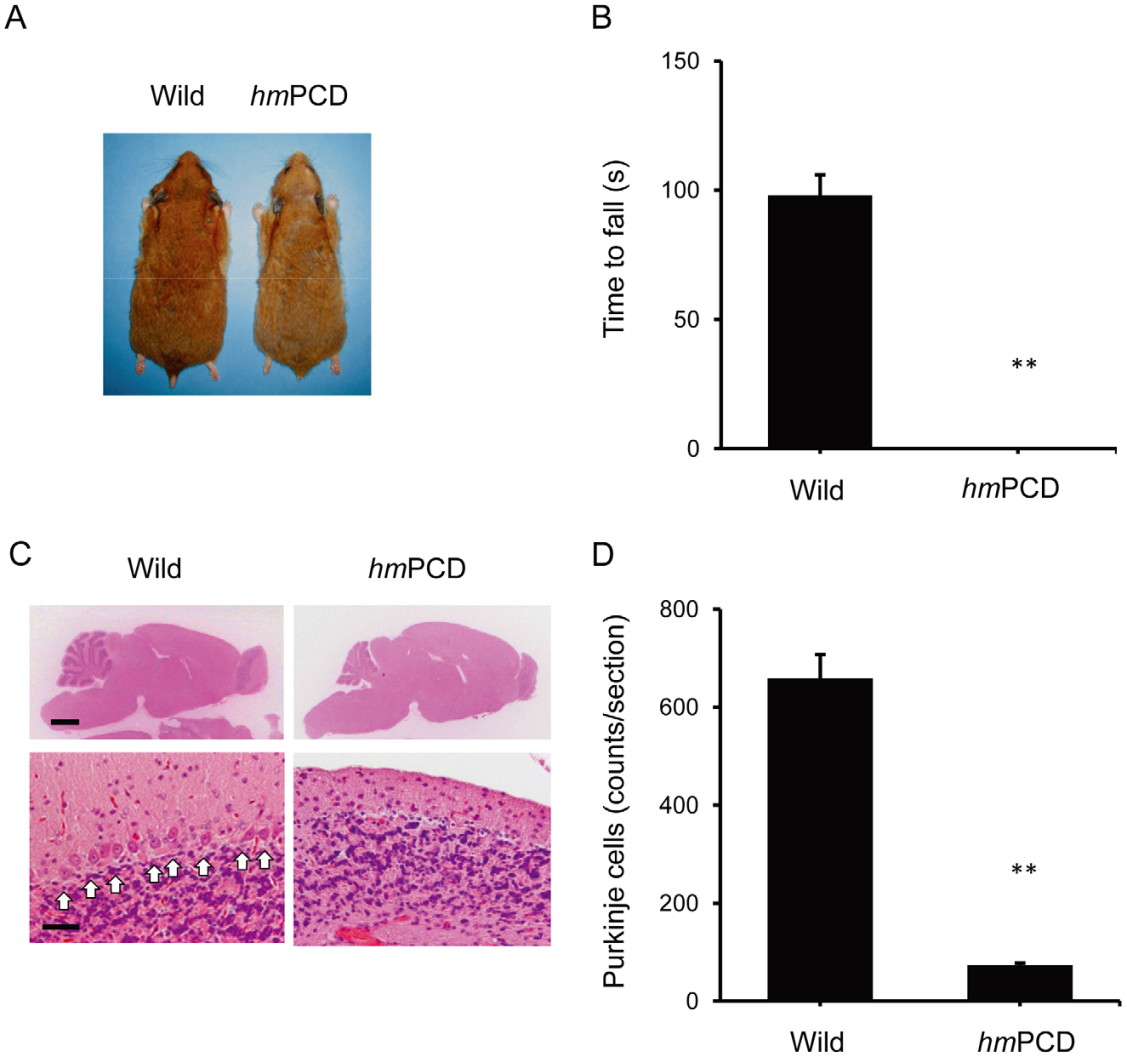
Overview of phenotype and behavioral disorders in the Purkinje cell-degenerated ataxic mutant hamster (*hm*PCD). (A) Representative appearance of a male *hm*PCD and a wild type control at 18 months of age. (B) Rota-rod testing of *hm*PCDs and age-matched wild type controls. The time spent on the rotating rod (6 rpm) is shown. (C) H&E-stained sections of the cerebellum from a *hm*PCD and a wild type control at 18 months of age. Purkinje cells are indicated with arrows. (D) Purkinje cell counts in cerebellar mid-sagittal sections from *hm*PCDs and wild type controls at 18 months of age. Bars: 2 mm and 50 µm for low and high power fields, respectively. Graphs show the mean ± SEM of 6 hamsters (3 males and 3 females). **P<0.01 vs. wild type control.

Given the effects of NK-4 on neurotoxicity *in vitro*, we evaluated NK-4 for treatment of neurodegeneration *in vivo* using an ataxic animal model in Syrian hamster characterized by Purkinje cell degeneration (*hm*PCD). Homozygous mutants were viable at birth and indistinguishable in appearance from normal controls (Fig. 7A), but grew up to be smaller than age-matched wild type hamsters. The mutants had a normal life span [23], but showed motor and behavioral deficits, including abnormal tremor and unusual falling. In a rota-rod test, all homozygous mutants fell after grasping the rod only briefly (Fig. 7B). Histological analysis using H&E staining showed marked atrophy of the cerebellar cortical region of the mutants at 48 weeks of age (Fig. 7C). Concomitantly, in the cerebellar cortex, a large reduction in the number of Purkinje cells was observed in the mutant brain (Fig. 7D).

### Effect of NK-4 on cerebellar ataxia in *hm*PCD

**Figure 8:**
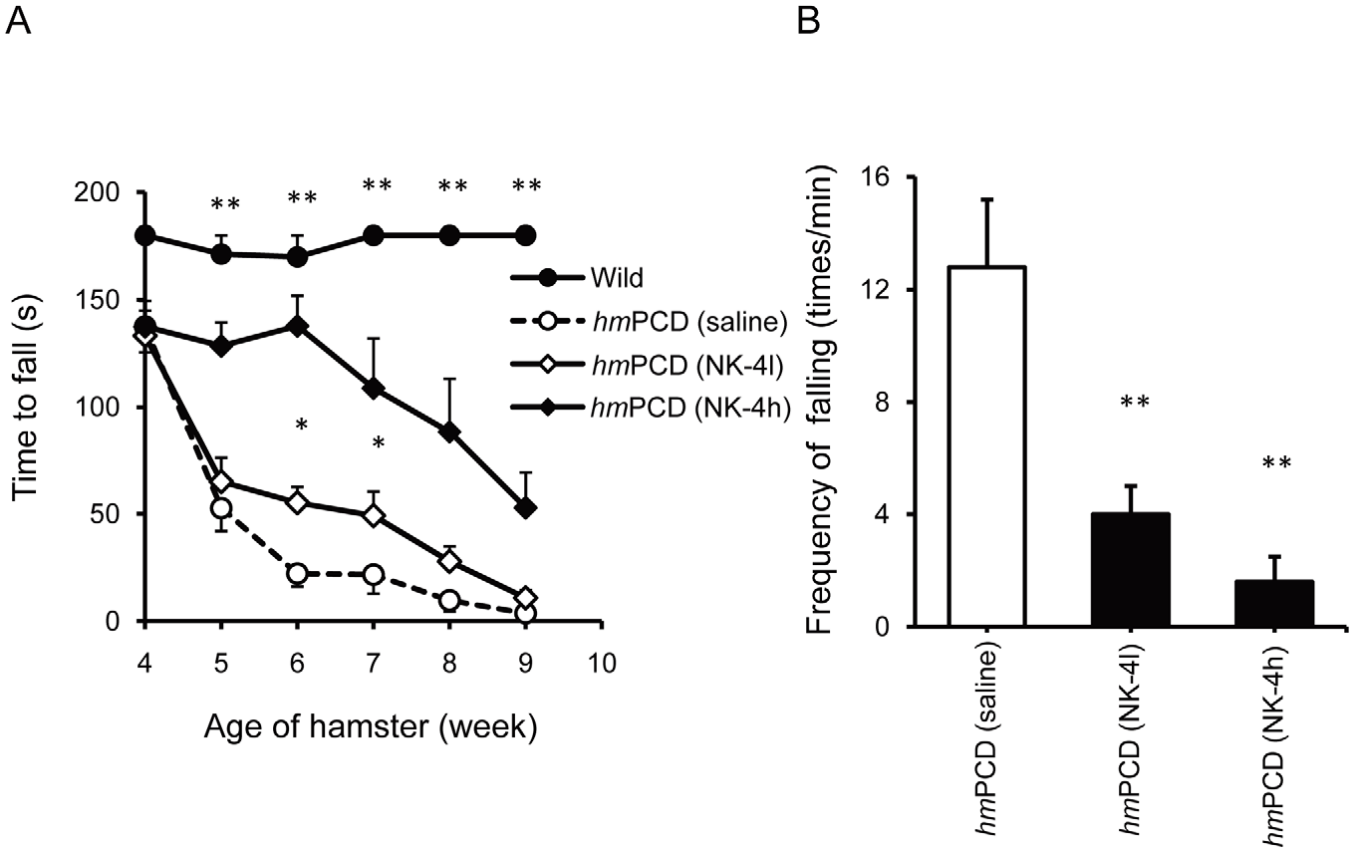
Effects of NK-4 on motor coordination in *hm*PCDs. (A) Effect of NK-4 on motor performance in the rota-rod test. Animals were tested weekly for the ability to remain on the rotating rod, and the time spent on the rod is shown. (B) Effect of NK-4 on frequency of falling in *hm*PCDs. Spontaneous falling of each animal was counted for 60 s and is presented as the falling frequency. Values are the mean ± SEM of 5 animals per group (2 males and 3 females). *P<0.05, **P<0.01 vs. vehicle-treated *hm*PCD.

Animals were treated with 20 or 100 µg/kg NK-4 once a day for 7 weeks, beginning at 3 weeks of age. We designed this dose schedule based on preliminary experiments for dosing of NK-4. In general, the animals remained in good health throughout the study. Motor discoordination of ataxic and non-ataxic animals was evaluated with rota-rod testing. Preliminary experiments showed that mutant animals had a moderate level of motor discoordination at 4 weeks of age, and that the disease progressed over time. As shown in Fig. 8A, a low dose of NK-4 (20 µg/kg) elicited a moderate, but significant, effect in attenuating deterioration of motor function that lasted for 2 weeks after 3 weeks of administration. A high dose of NK-4 (100 µg/kg) gave a strong effect that lasted for the whole test period. However, the rotation time for animals in the treated group was not comparable to the wild type controls, which were able to stay on a rotating rod for the maximum testing period (180 s). The effect of NK-4 on motor discoordination of *hm*PCDs was similarly observed in an inclined plane task [39] (data not shown).

At the end of the study (10 weeks of age), the motor ability of NK-4 treated animals was evaluated by counting the falling frequency. The *hm*PCDs began to fall frequently in their cage from around 7 weeks of age and the frequency of falling increased with age. Animals treated with low and high dose NK-4 showed a significant reduction in falling frequency compared to saline-treated controls (Fig. 8B). These observations show that NK-4 is effective for treating motor discoordination of cerebellar ataxia in the *hm*PCD model.

### Effect of NK-4 on cerebellar atrophy in *hm*PCD

**Figure 9:**
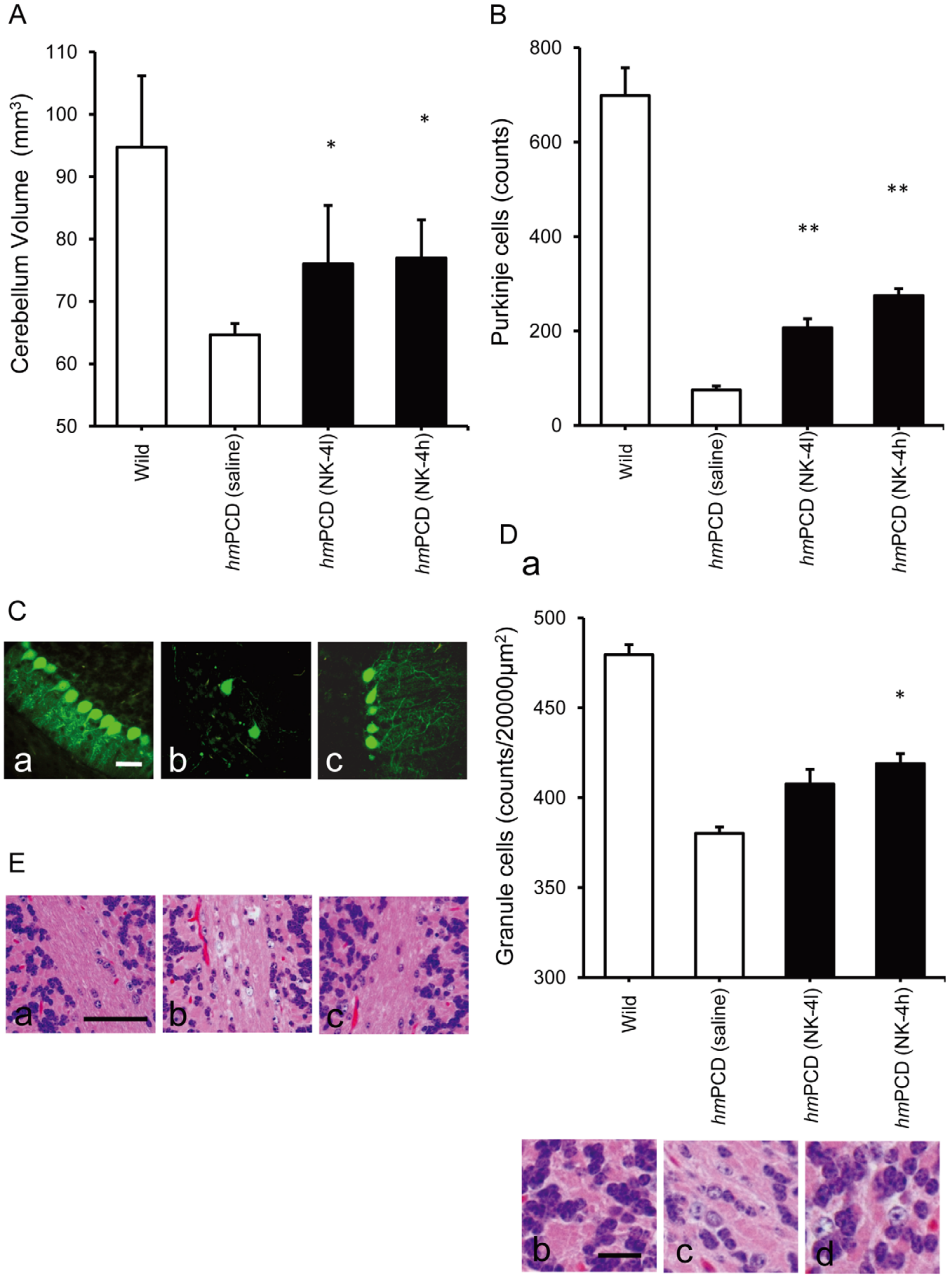
Effects of NK-4 on cerebellar atrophy and histopathology in *hm*PCDs. (A) Effect of NK-4 on cerebellar size in *hm*PCDs. Brains from 10-week-old animals were photographed from a constant distance and the cerebellar volume was calculated. (B) Effect of NK-4 on the Purkinje cell frequency in *hm*PCDs. Purkinje cells in the Purkinje cell layer were counted in the mid-sagittal section of the cerebellum from *hm*PCDs. (C) Immunohistochemistry using an anti-calbindin antibody on cerebellum sections for wild type (a), saline-treated *hm*PCD (b), and high dose NK-4 (100 µg/kg)-treated *hm*PCD (c). Bar: 30 µm. (D) Granule cell density in the cerebellum from *hm*PCDs and wild type controls. H&E stained sections of cerebellum cortex from *hm*PCDs and wild type controls were photographed and counted for granule cells in an area of 20000 µm^2^ (a). Representative images for wild type (b), saline-treated *hm*PCD (c), and high dose NK-4-treated *hm*PCD (d). Bar: 10 µm. Graphs show the mean ± SEM of 6 hamsters at 10 weeks of age (3 males and 3 females). *P<0.05, **P<0.01 vs. *hm*PCD control. (E) Attenuation of cerebellar demyelination in the white matter. Representative microphotographs of cerebellar white matter for wild type (a), saline-treated *hm*PCD (b), and high dose NK-4-treated *hm*PCD (c). Bar: 50 µm.

We next examined the cerebellar sizes in the animals (Fig. 9A). The mutants had severe cerebellar atrophy and volumetric reduction (about 60% of age-matched wild type animals) at 10 weeks of age. In contrast, the volume of the cerebrum did not differ significantly between mutants and wild type controls (data not shown). Animals treated with low or high doses of NK-4 had a significantly larger cerebellum volume compared with saline-treated controls. Thus, NK-4 was effective in ameliorating motor function and retaining cerebellar volume, which suggests that maintenance of Purkinje cells function might be the key step for achieving recovery of motor function.

H&E staining of the cerebellar cortex sections revealed a large reduction in the number of cerebellar Purkinje cells in the brain of *hm*PCDs at 10 weeks of age (Fig. 9B), which was further confirmed by immunostaining of Purkinje cells with anti-calbindin antibody (Fig. 9C). The number of calbindin-positive cells was well correlated with the number of Purkinje cells (Fig. 9B) and treatment with NK-4 (both low and high doses) significantly attenuated the loss of calbindin-positive cells compared with saline-treated controls. The dendrites of surviving calbindin-positive cells in slices from saline-treated *hm*PCDs had degenerated and weak features that made them difficult to find. In contrast, calbindin-positive cells in slices from high dose NK-4-treated *hm*PCDs had significantly longer and arborized dendrites (Fig. 9C).

In the brain of *hm*PCDs, the cerebellar granule cell density was moderately (∼80%) reduced compared to wild type controls, and the granule cells in the cerebellum from control *hm*PCDs showed atrophic feature (Fig. 9D). Effects of NK-4 against neuronal degeneration were also found in this context. NK-4 dose-dependently prevented the reduction of granule cells and attenuated the granule cell atrophy. Typical examples of H&E-stained sections of cerebellar white matter are shown in Fig. 9E. Saline-treated ataxic mutants displayed moderate levels of demyelination in cerebellar white matter (5/5 animals), while fewer animals showed demyelination after treatment with low and high doses of NK-4 (NK-4l; 4/5 animals, NK-4 h; 1/5 animals).

## Discussion

Chronic neurodegenerative diseases are characterized by a selective loss of specific neuronal populations over a period of years. Although the underlying causes of most neurodegenerative diseases are unclear, the loss of neurons and neuronal contacts is a critical feature of the disease pathology. Development of a compound that regulates neuronal cell death is a plausible therapeutic strategy to slow or halt the progression of neurodegenerative disease. In this study, we showed that NK-4, a photosensitizing cyanine dye, had neurotrophic effects, and found that promotion of proliferation and neurite-outgrowth of PC12 cells by NK-4 was dependent on activation of PI3K-Akt and occurred independently of Trk. Subsequently, we investigated the effects of NK-4 in an animal model of cerebellar ataxia attributable to Purkinje cell degeneration, and found that NK-4 effectively attenuated motor discoordination and prevented degeneration of cerebellar Purkinje and granule cells.

More than 20 cyanine dyes have been shown to exhibit neurotrophic effects, but NK-4 is one of the most potent agents for promotion of growth and differentiation of PC12 cells. Radical scavenging by NK-4 was also more effective than that of other dyes, and this led us to examine NK-4 for treatment of neurodegerative disease. In the present study, NK-4 clearly promoted PC12 cell growth and neurite-outgrowth primed by NGF. Consistent with these observations, ascorbate, a major H_2_O_2_ scavenger, also promotes NGF- or cAMP-primed neurite-outgrowth in PC12 cells [40], [41]. A superoxide scavenger, mitochondrial manganese superoxide dismutase (SOD), has also been reported to induce neurite-outgrowth in PC12 cells, with long term NGF-induced activation of extracellular signal-related kinase (ERK1/2), which regulates neurite-outgrowth in PC12 cells, required for SOD activation [42]. NGF exerts a neuritogenic effect by changing mitochondrial metabolism by reduction of ROS produced by mitochondria and stabilization of the electrochemical gradient. These observations suggest that NK-4 promotes neurite-outgrowth in PC12 cells by reduction of ROS via induction and maintenance of long term activation of ERK1/2.

Direct scavenging of free radicals may be one mechanism through which NK-4 exerts neuronal protection against oxidative damage. NK-4 protected PC12 cells from oxidative toxicities induced by H_2_O_2_ and 6-OHDA in a significant and dose-dependent manner (Fig. 2AB). However, NK-4 did not scavenge H_2_O_2_, which suggests that NK-4 can detoxify radical species generated from H_2_O_2_. ROS play pivotal roles in the pathogenesis of neurodegenerative diseases, since increased formation of superoxide radicals leads to increased of production of H_2_O_2_ due to (SOD) activity. In living cells, H_2_O_2_ is a major underlying cause of oxidative damages [43], and is readily converted to highly reactive hydroxyl radicals via a Fenton reaction in the presence of iron ions [44], [45]. These are the most oxidizing radicals known in biological systems and may be involved in neurodegeneration. NK-4 is a highly effective free radical scavenger for hydroxyl and peroxy radicals and a moderate scavenger for superoxide [18]; therefore, NK-4 may detoxify these radical species before they cause cellular damage.

We investigated whether TrkA and its downstream signal transduction pathways were involved in NK-4-induced proliferation and neurite-outgrowth in PC12 cells. TrkA is the cell surface receptor through which NGF mediates neuronal survival and differentiation in PC12 cells [19]. K252a, an inhibitor of tyrosine autophosphorylation of Trks, inhibited proliferation of PC12 cells induced by NGF, but did not inhibit that induced by NK-4 (Fig. 3A). This result suggests that neurotrophic activity by NK-4 is not mediated by TrkA or other K252a-sensitive molecules in PC12 cells. Consistent with this observation, K252a also dose-dependently inhibited NGF-induced neurite extension in PC12 cells, but did not effectively inhibit that induced by NK-4 primed with NGF (Fig. 3B). This indicates that neuronal cell growth induced by NK-4 occurred independently of Trk activation, but that neurite-outgrowth in PC12 cells required activation of K252a-sensitive Trk receptors. In contrast, LY294002, a specific inhibitor of PI3K, affected cell growth induced by both NK-4 and NGF (Fig. 4A) and inhibited neurite-outgrowth induced by NGF and NK-4 primed with NGF (Fig. 4B) in PC12 cells. These results suggest that PI3K is an essential regulator of NK-4-mediated effects. NK-4 also induced phosphorylation of Akt, a downstream regulator of PI3K, in PC12 cells (Fig. 5), which indicates that the PI3K-Akt signaling pathway is affected by NK-4.

In agreement with the activation of survival signaling pathways, we also showed an effect of NK-4 on SAPK/JNK activation induced by H_2_O_2_. This pathway is relevant to neuronal death because neurons undergo apoptosis through the SAPK/JNK pathway. SAPK/JNK is also activated by neurotrophic factors such as NGF, and this process is a key for neurotrophic factor-mediated neuronal apoptosis. NGF induces SAPK/JNK activation within 1 day of treatment [46], whereas NK-4 (∼6.3 µM) did not induce SAPK/JNK activation for up to 3 days (data not shown). This result is consistent with our previous observation that NK-4 mediated signaling is independent of neurotrophin receptors because SAPK/JNK is a downstream effector of Trks [46]. In the present study, NK-4 inhibited H_2_O_2_-induced activation of SAPK/JNK in PC12 cells (Fig. 6), and the viability of PC12 cells subjected to H_2_O_2_ toxicity with or without treatment with NK-4 was negatively correlated with SAPK/JNK phosphorylation. Thus, NK-4 might activate the survival signaling cascade and inhibit the death signaling cascade independently through a Trk-mediated pathway, and this may provide protection against the neurotoxic environment. We did not determine whether the low affinity p75 neurotrophin receptor for NGF might also be a target of NK-4, but this appears unlikely because selective activation of p75 NTR by NGF stimulates JNK activity and apoptotic cell death [47], in contrast to the inability of NK-4 to activate JNK and apoptosis.

The effect of NK-4 against neurodegeneration was confirmed in an animal model of ataxia. We recently showed that NK-4 attenuated brain injury and improved motor function in a rat middle cerebral artery occlusion model [18]. This study indicated the efficacy of NK-4 on the central nervous system (CNS); however, the administration route remained in question because disruption of the blood-brain barrier in this model [48] might change the permeability of NK-4. Therefore, we used a genetic animal model of ataxia (*hm*PCD) to evaluate the effect of NK4 on neurodegenerative disease. This animal model reflects the pathogenesis of human spinocerebellar ataxia [24], and exhibits extensive cerebellar atrophy, including a substantial loss of Purkinje cells and a mild reduction of granule cells, along with decreased thickness of the molecular layer and internal granule layer. In contrast, the cerebral hemisphere has no altered features and no neurodegeneration [23]. The gene responsible for this cerebellar atrophy is *Nna1*, which encodes a putative nuclear protein containing a zinc carboxypeptidase domain initially identified by its induction in spinal motor neurons during axonal regeneration [25]. Blood-brain barrier permeability in this animal model is unlikely to be affected by Nna1. In the *hm*PCD model, 20 or 100 µg/kg NK-4 was effective in attenuating motor discoordination and Purkinje cell loss with no toxic effects. This suggests that NK-4 can attenuate neurodegeneration in the CNS via a peripheral route of administration. The dose of NK-4 used in this study was equivalent to that used in the rat middle cerebral artery occlusion model. From these observations, NK-4 would cross the blood-brain barrier and acted directly on degenerating neurons and protected against cell damage. The concentration of NK-4 in the cerebellum of *hm*PCDs remains in question. In our preliminary study, NK-4 penetrated into the cerebellum, and was detected at nM levels in the cerebellum from 15 min after intraperitoneal administration in hamster (100 µg/kg) using high-performance liquid chromatography detection system. The concentration found in cerebellum was comparative to that found in plasma. These results were consistent with the *in vitro* observations that nM levels of NK-4 was sufficient to exert its neurotrophic effects.

Purkinje cells are susceptible to ischemic damage because of their reduced capacity to isolate glutamate and reduced ability to generate energy during anoxia [49]. Granule cells are also vulnerable to a variety of toxins that decrease glutathione levels and this make the cells more vulnerable to DNA and other cellular damage from ROS [50], [51]. Direct scavenging of free radicals by NK-4 may protect these cells. In addition, NK-4 seems to activate survival signaling pathways in degenerating cerebellar neurons. This is supported by the protection of Purkinje and granule neurons from cell death by activation of a PI3K-Akt dependent mechanism [52], [53]. Calbindin-positive Purkinje cells in NK-4-treated animals possessed longer and well-branched neurites, in contrast to the feeble dendrites in saline-treated *hm*PCDs, which suggests functional differences (Fig. 9C) between neurons in these animals. This might provide a better explanation of the improved motor coordination in the NK-4-treated animals, rather than dependence on the Purkinje cell number. The number of granule neurons in treated animals was also higher than that in the saline-treated controls (Fig. 9D). This result is consistent with the observation that Purkinje cells provide critical trophic support to developing cerebellar granule neurons [54]. Granule cells are the most abundant and provide input to the Purkinje cells, whose axons are the sole output from the cerebellar cortex and provide input to multiple target cells. These observations suggest that the attenuation of cerebellar atrophy by NK-4 may be a result of restoration of these two main cell types.

In summary, we conclude that NK-4 exerts neurotrophic activity both *in vitro* and *in vivo*, and that this activity may be mediated partially through direct free radical scavenging, especially of hydroxyl and peroxy radicals. Analysis of signaling events revealed that the PI3K-Akt survival signaling cascade was triggered by NK-4 without Trk activation. Results in an ataxic animal model demonstrated the preclinical efficacy of NK-4 and suggested its potential utility as a treatment for neurodegenerative disease. Assessment of NK-4 in human neurodegenerative therapy will require further studies. A safety evaluation and the pharmacokinetics of NK-4 are currently being investigated.
